# Evaluation of commercial doses of a feed additive and silymarin on broiler performance with and without CCl_4_-induced liver damage

**DOI:** 10.1016/j.psj.2024.103567

**Published:** 2024-02-18

**Authors:** Mabel Barreiro Carpio, M. Alejandro Valdes-Pena, Daniel A. Molina, Sandra E.J. Espinoza Cabello, Carlos A. Sialer Guerrero, Giovanna Cribillero, Katherine F. Vargas Coca, Eliana Icochea

**Affiliations:** ⁎ R&D Department, Ilender Perú S.A., Lima, Peru; † Department of Chemistry, NC State University, Raleigh, NC; ‡ School of Veterinary Medicine, Universidad Nacional Mayor de San Marcos, San Borja, Lima, Peru; § Department of Poultry Science, Mississippi State University, Mississippi State, MS

**Keywords:** phytogenic feed additive, toxin, poultry, productive performance

## Abstract

Improving productive performance is a daily challenge in the poultry industry. Developing cost-effective additives and strategies that improve performance in antibiotic-free poultry production is critical to maintaining productivity and efficiency. This study evaluates the influence of a commercially available phytogenic feed additive (**CA-PFA**, that comprises silymarin, betaine and curcumin extracts as main ingredients) and silymarin on commercial broilers' productive performance and liver function with and without carbon tetrachloride (**CCl_4_**)-induced liver damage. The experiment was conducted in a completely randomized design, with six treatments, eight replicates, and eight birds per replicate in 18 one-day-old male broilers (Cobb Vantress 500) each; under a 3 × 2 factorial arrangement (3 diets x 2 levels of CCl_4_, 0 and 1 mL/kg body weight orally). The experimental treatments included 3 diets, commercially recommended doses of CA-PFA (500 mg/kg of feed; this dose provides 70 mg/kg of silymarin, besides the other active ingredients included in the formulation), silymarin (250 mg/kg of feed, containing 28% of active ingredient; this dose provides 70 mg/kg of silymarin as active ingredient) and an additive-free basal diet as a control. A standard commercial silymarin was used as a reference due to its well-known and extensively studied hepatoprotective properties that can mitigate the negative effects of CCl_4_ in the liver. The data were analyzed as a 2-way ANOVA, and the means showing significant (*P* ≤ 0.05) differences were then compared using the Post-Hoc Tukey HSD test. No interaction was detected between factors. Exposure to CCl_4_ had a noticeable detrimental effect on alertness, productive performance, and liver function of broilers without a significant increase in mortality. Including CA-PFA in the diet improved productive performance compared to the basal diet from day 21 to the end of the trial, on day 42. While no influence in feed intake was detected for any treatment, CA-PFA improved body weight gain (BWG) and feed conversion ratio (FCR) significantly (*P* < 0.05) from day 21 to the end of the trial in healthy and CCl_4_-exposed birds. The results show that CA-PFA supplementation improves performance parameters in broilers with and without CCl_4_-induced liver damage, when compared to a basal diet and the addition of a standard commercial silymarin product.

## INTRODUCTION

Improving productive performance using cost-effective approaches is a daily challenge in the poultry industry, which produces low-cost nutritious foods for human consumption. Adding phytogenic feed additives (**PFA**) into the diet has become a frequent solution to boost production, especially after the implementation of restrictions and bans on the use of antibiotic growth promoters in different countries (Rahman et al., 2022). There are many well-studied PFAs with proven benefits, but the doses reported in the literature are usually higher than the commercial doses recommended in the poultry industry (Rahman et al., 2022; Surai and Surai, 2023; El-Sabrout et al., 2023), generating a disconnection between the reported benefits and the industry experience. Therefore, it is key to study the efficacy of commercial products or promising feed additives at commercially recommended levels. This study assesses the impact of the recommended dose of a commercially available PFA (**CA-PFA**) on broiler performance and whether that effect is linked to improved liver function. The CA-PFA selected (inaplus3, Ilender Peru SA) claims to optimize broiler performance by improving overall health and metabolic activity. CA-PFA contains a mixture of different PFAs, including milk thistle extract (silymarin), turmeric extract, and betaine hydrochloride.

Silymarin, betaine, and turmeric extracts have been extensively studied because of their benefits, which have been summarized in many reviews (Yarru et al., 2009; Akhavan-Salamat and Ghasemi, 2016; Zaker-Esteghamati et al., 2020; Nawab et al., 2020; Mishra et al., 2021; Abd El-Hack et al., 2021; Abd El-Ghany and Babazadeh, 2022; Alagawany et al., 2022; Ugo et al., 2022; Elnesr et al., 2023; Gana et al., 2023; Guerrini and Tedesco, 2023; Surai and Surai, 2023; Sureshbabu et al., 2023). Adding these active ingredients to livestock and poultry feed improves metabolism, reproduction, immunity, nutrient digestibility, fat metabolism and can alleviate chronic liver injuries and mitigate the detrimental effects of heat stress, improving overall health and favoring animal growth and development. However, there is still conflict regarding the minimum doses required to observe statistically significant effects. Supplementing poultry diets with curcumin at 200 mg/kg of feed or higher improves productive performance (Rajput et al., 2013; Sureshbabu et al., 2023). Betaine doses frequently used in studies that report statistically significant improvement in productive performance are 500 mg/kg of feed or higher (Sakomura et al., 2013; Yang et al., 2022; Zaki et al., 2023). Silymarin inclusion in poultry diets improves broiler performance, however, results at low doses (100–200 mg/kg feed) are inconsistent, with some studies showing performance improvement (Armanini et al., 2021; Shanmugam et al., 2022; Surai and Surai, 2023) while other studies report statistically non-significant differences, especially if broilers are grown under normal physiological conditions (Schiavone et al., 2007; Blevins et al., 2010; Sakamoto et al., 2018; Lukanov et al., 2019; Zaker-Esteghamati et al., 2020). All the doses that have been confirmed as effective for these additives are higher than commercially recommended doses because of profitability reasons.

Among the different components present in CA-PFA, silymarin is one of the most well-studied and has been proposed previously as a natural hepatoprotective comparison standard (Dhiman and Chawla, 2005). Therefore, we decided to include silymarin as a control treatment in the present study and explore more deeply the potential hepatoprotective activity of CA-PFA as the reason behind its effectiveness in improving broiler productive performance.

The liver is a vital organ that plays a crucial role in metabolism through its many complex physiological functions, including excretion, detoxification, and nutrient modification and absorption. With the steadily increasing intensive farming industry, the liver is frequently damaged by chemical contaminants such as mycotoxins and misbalances present in the diet (Rozenboim et al., 2016; Haque et al., 2020; Guerrini and Tedesco, 2023). An impaired hepatic function, even without clinical signs, can affect the profitability of the poultry industry by reducing nutrient absorption, which ultimately affects the feed conversion ratio (**FCR**) and reduces the return on investment in the feed. Preventing and treating hepatic damage is an effective mechanism to maintain and improve productive performance. In this work, we investigate if CA-PFA hepatoprotective effect is the cause of its impact on productive performance. For that, we evaluated the influence of CA-PFA, silymarin, and an additive-free diet on the productive performance of broilers with and without liver damage induced by CCl_4_.

CCl_4_ is a chemical agent frequently applied to induce liver toxicity in experimental models that aim to study hepatoprotective properties using *in vivo* approaches, including broilers (Weber et al., 2003; Baradaran et al., 2019). At low doses, CCl_4_ selectively causes dose-dependent toxicity in liver cells without strongly affecting other metabolic functions (Rao et al., 1997; Weber et al., 2003; Sonkusale et al., 2011). Overall, the CCl_4_-induced liver damage model is an effective, reproducible strategy to induce hepatotoxicity with low mortality and, therefore, is extremely useful in studying hepatoprotective activity. (Sonkusale et al., 2011; Wang et al., 2013; Joseph et al., 2014; Baradaran et al., 2019).

The present work explores the CA-PFA influence on broiler productive performance with and without induced liver damage. CA-PFA components (silymarin, turmeric, and betaine) present many proven benefits, like hepatoprotective activity, but the documented effective doses for the individual components are higher than the amount CA-PFA provides to the diet at the recommended dose (250–500 mg/kg of feed). Hence, the main objective of the present study was to determine if CA-PFA supplementation is more effective than silymarin at improving broilers' growth performance, at commercially recommended doses, under standard growing conditions and with induced liver damage.

## MATERIALS AND METHODS

### Experimental Design, Birds, and Management

The animal use and care procedures followed the Guide for the Care and Use of Agricultural Animals in Research and Teaching (FASS, 2010). All procedures concerning animal treatments and experiments were reviewed and approved by the Institutional Animal Care and Use Committee at the School of Veterinary Medicine of the Universidad Nacional Mayor de San Marcos, Lima, Perú (approval code CEBA 2020-10). The experiment was conducted in a completely randomized design, where 864 one-day-old male broilers (Cobb Vantress 500) were randomly divided into 6 treatment groups with 8 replicates of 18 birds each. The birds were vaccinated at hatching against Marek´s disease and had ad libitum access to water and feed during the entire experimental period. A three-phase feeding program without any medication or anticoccidials was used, with starter (1–21 d), grower (22 –37 d), and finisher diets (38–42 d) (Table 1).

**Table 1:** Composition and nutrient levels of the basal diets used during starter, grower, and finisher stages. Additives tested (silymarin and CA-PFA) were added on top to the corresponding diets after they were formulated.

Items	Content per stage		
	Starter (1–21 d)	Grower (22–37 d)	Finisher (38–42 d)
Ingredients (%)			
Corn	65.70	67.58	70.82
Soybean meal	30.00	26.00	23.00
Soybean oil	0.00	2.40	3.00
Dicalcium phosphate	1.75	1.65	1.30
Calcium carbonate	1.22	1.12	1.10
Salt	0.24	0.21	0.21
DL-methionine	0.14	0.11	0.10
L-Lysine	0.29	0.23	0.22
Sodium bicarbonate	0.18	0.23	-
Vitamin and mineral premix^1^	0.10	0.10	0.10
Choline-Cl 60%	0.05	0.05	0.05
Mycotoxin binding agent	0.25	0.25	-
L-Threonine	0.06	0.06	0.10
Antioxidant	0.02	0.02	-
Total	100.00	100.00	100.00
Nutrient levels (% as fed)^2^			
ME (kcal/kg)	2899.94	3074.98	3159.92
Crude Protein	18.48	16.78	15.79
Crude Fiber	3.10	2.95	2.88
Ca	0.96	0.88	0.79
Total Phosphorus	0.66	0.63	0.55
Nonphytate phosphorus	0.42	0.40	0.33
DLys	1.14	0.99	0.92
DMet	0.40	0.34	0.33
DMet+DCys	0.66	0.58	0.56

1 Provided per kg of product: Vitamin A - 12000 IU; vitamin D3 - 5 000 000 IU; vitamin E - 30 000 IU; thiamine - 2 g; riboflavin - 10 g; pyridoxine 3 g; vitamin B12 - 0.015 g; pantothenic acid - 11 g; niacin - 30 g; biotin - 0.15 g; folic acid -2 g; Iron - 50 g; zinc - 80 g; manganese - 80 g; iodine - 1 g; copper - 12 g; selenium - 0.30 g; cobalt - 0.4 mg

2 The nutrient levels were calculated values.

The experiment was conducted under a 3 × 2 factorial arrangement, where factors included three different treatments and CCl_4_ (0 and 1 mL/kg BW orally, which based on reagent density, corresponds to 1.59 g/kg BW) administered every third-day during 15 to 28 d of trial. Treatments consisted of basal diet, standard commercial silymarin 28% (dose 250 mg/kg of feed, containing to 70 mg/kg of silymarin), and CA-PFA (inaplus3, from Ilender Peru S.A., Lima, Peru) (dose 500 mg/kg of feed, which provides to the diet 70 mg/kg of silymarin) as feed additives. Inaplus3 composition, as described by the supplier, combines *Silybum marianum* or silymarin (this active ingredient is extracted from *Milk Thistle* and contains silybin and isosilybin), betaine (used as hydrochloric salt) and turmeric extract (rich in curcumin, main bioactive compound found in *Curcuma Longa Linn* plants, specifically in the roots). Both treatments (silymarin and CA-PFA) provided the same amount of silymarin as an active compound, and the minimum accepted analyzed content of this active ingredient was 65 mg/kg the different treatments and diets used. The diets were fed to birds throughout the experiment ad libitum. CCl_4_ (CAS 56-23-5) was purchased from Nexgen Chemical, batch number NEX3615, content > 98%), relative density (to water =1) 1.59 g/mL.

### Parameters Evaluated and Sample Collection

Productive performance parameters (body weight (**BW**), body weight gain (**BWG**), and feed intake (**FI**)) of each group were registered every week during the whole experiment. Mortality was recorded daily. The FCR was calculated as BWG divided by FI after correcting for mortality. The total evaluation period lasted 42 d.

On day 28 (at the termination of CCl_4_ dosing) and on day 42 of age (at the termination of all treatments), one representative bird per group (n = 8) was randomly selected, weighed, and euthanized by cervical dislocation following AVMA guidelines for the euthanasia of animals (Leary and Johnson, 2020). Blood and liver were extracted from each bird to obtain histopathological samples and to determine liver relative weight. The liver was dissected and weighed without removing the gallbladder, expressing the results as the relative percentage of the liver with respect to the body weight of the bird.

Histopathological liver samples obtained were fixed in 10% formalin for 48 h and dehydrated with ethanol. Then the tissue was embedded in paraffin that was cut into 5 to 6 μm slices. Sections were subjected to hematoxylin and eosin (**H&E**) staining and then observed under an optical microscope according to previously reported procedures (Cherian et al., 2002). Histological assessment of the tissue was performed by a pathologist blinded to the treatment groups.

Plasma samples were obtained by collecting 2 to 3 mL of blood from the branchial vein, following the guidelines described by Crespo and Shivaprasad (2015). Collected blood samples were poured into 5 mL sterilized vials and allowed to stand while inclined for 2 h. The separated serum was transferred to 1.5 mL vials and frozen. Plasma samples were submitted for analysis to measure biochemical parameters, specifically aspartate amino transferase (**AST**), alkaline amino transferase (**ALT**), gamma glutamyl transferase (**GGT**), alkaline phosphatase (**ALP**), total protein, and albumin. These parameters were estimated using diagnostic kits (Human Gesellschaft, Germany) through UV-Vis spectrophotometry using supplier guidelines.

### Statistical Analysis

The experiment was carried out in a completely randomized 3 × 2 design, with 6 experimental treatments, 8 groups within each treatment and 8 birds per group. Data were analyzed as a 2-way ANOVA, including the main effects of diet, challenge, and their interaction. Each group of eight birds was considered 1 experimental unit for the statistical analysis. The means showing significant (*P* ≤ 0.05) and trending (0.05 < *P* ≤ 0.10) differences in the ANOVA were then compared using the post-hoc Tukey HSD test.

## RESULTS

### Experimental CCl_4_-Induced Liver Toxicity Model

The groups that were not exposed to liver damage caused by CCl_4_ remained active and alert during the whole experiment, and no changes in behavior were noticed. After each CCl_4_ dose, the challenged birds presented a decrease in activity, but normal behavior was restored by the next day. CCl_4_ had a negative effect on productive performance without causing a significant increment in mortality. Therefore, the final viability during the experimental period was not significantly affected by the CCl_4_ administration. At the necropsy performed on day 28 (at the end of the challenge), the livers of birds exposed to CCl_4_ were slightly larger, greasier and paler in color. However, extremely severe lesions or hemorrhages were rarely detected during necropsies, and the biochemical parameters did not reflect extreme changes compared to control groups. According to the observations made during the trial and the clinical symptoms, CCl_4_-induced liver toxicity, at the applied dose, is a practical and straightforward method that affects liver function without atrophying its metabolism. Therefore, this model can be considered appropriate to evaluate the effect of hepatoprotective additives on the productive performance of birds with impaired hepatic function.

### Productive Performance

Before CCl_4_ administration, no significant differences were found in productive performance parameters (detailed data in Supporting Information, Table S1). The results obtained for productive performance during and after exposure to CCl_4_ are shown in Table 2. No interaction between factors (CCl_4_ treatment and feed additives used) was observed during the trial. Isolated effects for both factors studied were detected (*P* < 0.05) on BWG and FCR, showing the influence on the productive performance of the intoxicating capacity of CCl_4_ and the protective effect of the additives applied. No significant differences were found in FI, allowing an easier correlation between the improvements in BWG and FCR to the effects of the treatments used and not a difference in nutrient intake.

**Table 2:** Effect of CCl_4_ and additive used on accumulative BWG FI, and FCR.^1^

		BWG (g)				FI (g/bird)				FCR (g/g)			
		21 d	28 d	35 d	42 d	21 d	28 d	35 d	42 d	21 d	28 d	35 d	42 d
Challenge	Additive												
CCl_4_ 0 mL/kg BW	None	868^bc^	1471^b^	2315^b^	3147^bc^	1389	2508	4027	5694	1.601	1.708^bc^	1.742^bc^	1.810^ab^
	Silymarin	883^b^	1509^b^	2351^ab^	3240^ab^	1400	2556	4064	5760	1.585	1.694^bc^	1.729^bc^	1.778^ab^
	CA-PFA	925^a^	1581^a^	2428^a^	3315^a^	1402	2558	4073	5781	1.516	1.618^c^	1.679^bc^	1.745^b^
CCl_4_ 1 mL/kg BW	None	844^c^	1347^c^	2185^c^	3040^c^	1405	2520	4060	5730	1.666	1.871^a^	1.859^a^	1.886^a^
	Silymarin	848^bc^	1393^c^	2207^c^	3057^c^	1376	2487	3998	5669	1.624	1.787^ab^	1.813^ac^	1.857^a^
	CA-PFA	878^bc^	1460^b^	2316^b^	3207^ab^	1369	2479	4021	5744	1.561	1.698^bc^	1.736^bc^	1.794^b^
	*SEM (n = 8)*	*24.4*	*44.4*	*65.0*	*97.7*	*82.8*	*121.7*	*153.0*	*166.2*	*0.109*	*0.094*	*0.081*	*0.081*
Main effects													
CCl_4_	0 mL/kg BW	892^a^	1520^a^	2364	3234^a^	1397	2540	4055	5745	1.568	1.674^b^	1.717^b^	1.778^b^
	1 mL/kg BW	857^b^	1400^b^	2236	3102^b^	1383	2495	4026	5714	1.617	1.786^a^	1.803^a^	1.846^a^
	*SEM (n = 24)*	*31.1*	*63.4*	*82.1*	*118.9*	*80.1*	*118.2*	*148.1*	*162.7*	*0.112*	*0.107*	*0.088*	*0.084*
Additive	None	856^b^	1409^b^	2250	3094^b^	1397	2514	4044	5712	1.633^a^	1.790^a^	1.801^a^	1.848^a^
	Silymarin	866^b^	1451^b^	2279	3149^b^	1388	2521	4031	5714	1.605^ab^	1.741^ab^	1.771^ab^	1.817^ab^
	CA-PFA	902^a^	1520^a^	2372	3261^a^	1385	2518	4047	5762	1.539^b^	1.658^b^	1.708^b^	1.770^b^
	*SEM (n = 16)*	*30.2*	*75.7*	*91.6*	*117.9*	*81.1*	*121.8*	*150.3*	*163.5*	*0.108*	*0.109*	*0.091*	*0.086*
*Effect (P-value)*													
CCl_4_		<0.001	<0.001	<0.001	<0.001	0.578	0.205	0.526	0.528	0.124	<0.001	<0.001	0.006
Additive		<0.001	<0.001	<0.001	<0.001	0.912	0.985	0.951	0.635	0.051	0.001	0.007	0.029
CCl_4_ x Additive		0.417	0.967	0.787	0.463	0.675	0.513	0.615	0.562	0.939	0.409	0.586	0.852

a–c Means in the same column with different superscripts are different (*P* < 0.05)

1 BWG: body weight gain; FI: feed intake; FCR: feed conversion ratio

Both additives, silymarin and CA-PFA, improved BWG and FCR since day 21 in birds with CCl_4_-induced liver damage, showing that including additives in the diets alleviated the CCl_4_ detrimental effects. The differences are not statistically significant when comparing the performance of challenged birds treated with standard commercial silymarin to the challenged birds consuming an additive-free diet (*P* > 0.05). On the other hand, challenged groups treated with CA-PFA showed a significant improvement in BWG and FCR compared to the other challenged birds treated with silymarin or consuming an additive-free diet. Interestingly, by day 28 (at the termination of CCl_4_ dosing), challenged birds treated with CA-PFA presented BWG and FCR similar to those of unchallenged broilers (*P* > 0.05) and showed a significant improvement (*P* < 0.05) when compared to challenged broilers consuming feed without additives. The differences continued to be significant until the end of the experiment, indicating that CA-PFA ameliorated the toxic effects of CCl_4_ on productive performance. On the other hand, silymarin did not show similar protective effects at the dose used. Challenged birds consuming silymarin presented performance parameters similar to untreated challenged control birds and significantly lower BWG and FCR than healthy birds with control feed.

Both additives used, silymarin and CA-PFA, have a beneficial effect on growth performance even when no hepatotoxic event is present. BWG and FCR of birds treated with CA-PFA showed a significant improvement compared to the control group from day 21 until the end of the experiment (*P* < 0.001). In the case of silymarin, the numerical improvement consistently detected was not statistically significant in the conditions of the present experimental design. Unchallenged birds consuming the diet supplemented with CA-PFA presented the highest BWG and the best FCR of all groups since day 21. When compared with unchallenged birds consuming silymarin, CA-PFA showed significantly higher BWG on days 21 and 28 (*P* < 0.05). By the end of the experiment, the difference became smaller (*P* = 0.186) but still consistent.

### Necropsy Examination and Liver Characteristics

**Figure. 1:**
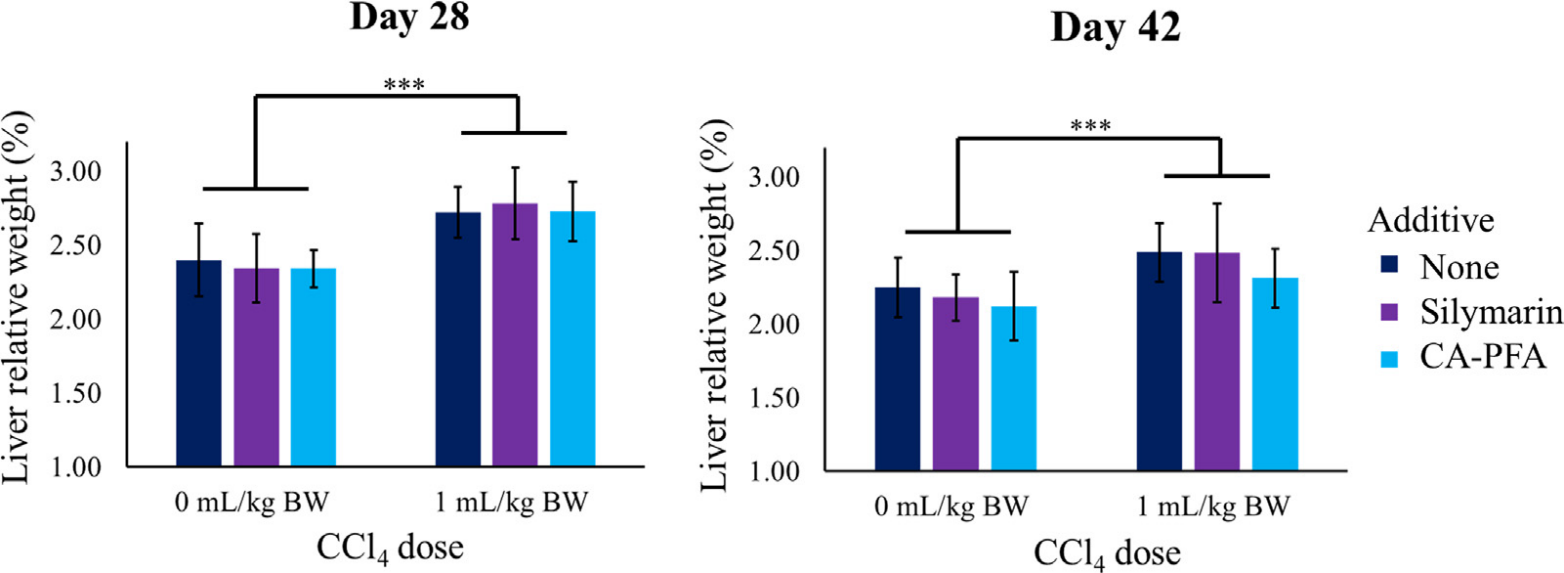
Effect of CCl_4_ doses and additives on liver relative weight at 28 and 42 d. Differences were only statically significant only for the factor CCl_4_ dose, not between independent treatments. *** corresponds to *P* < 0.001) Detailed data and *P* values for all comparisons can be found in Supporting Information Table S2.

**Figure 2:**
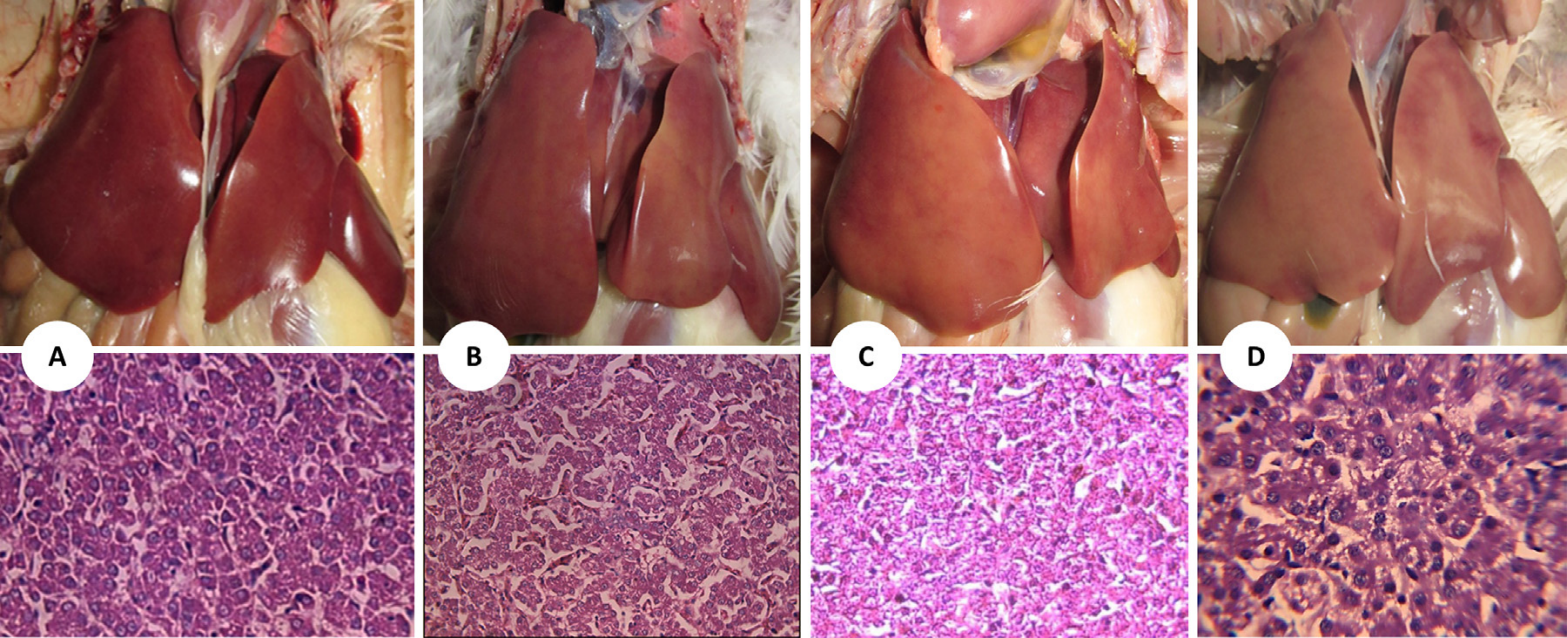
Representative images of the liver tissue showing different levels of fat infiltration and its corresponding images from liver histology by hematoxylin and eosin (H&E) staining. (A) healthy liver, flexible, shiny and red in color with no signs of lipid accumulation. (B) liver showing small fat drop accumulation. (C) liver tissue affected by a moderate fat infiltration. (D) representative livers showing severe lipid accumulation with big fat drops of steatosis form and distinct signs of hepatocyte dystrophy can be detected.

We observed slight pathological changes during the post-mortem examination performed on randomly selected birds by day 28 (end of CCl_4_ challenge). However, macroscopic evaluation of the organs showed individual rather than diet-related variations. Liver relative weights in broilers exposed to CCl_4_ were larger (Figure 1) and showed a greasier and paler appearance, especially on day 28 (Figure 2). Histological sections taken on day 28 from the livers of broilers exposed to CCl_4_ mainly showed a decrease in sinusoidal spaces, fatty degeneration, and liver congestion. Representative images of the liver tissue showing fat infiltration are presented in Figure 2, as are corresponding images from liver histology by H&E staining. By day 42, livers from challenged birds showed a healthier appearance compared to day 28, which was also validated by histopathological observations. Moreover, for the isolated effect of CCl_4_ exposure, the differences in relative liver weight between groups decreased (Figure 1) but remained significant (*P* < 0.05) (see Supplementary Information, Table S2 for further details).

### Biochemical Parameters

Results obtained for biochemical parameters are shown in Table 3. No isolated effects in connection with adding additives were detected on either day 28 or 42. On the other hand, CCl_4_-induced liver damage was found to slightly increase the concentration of ALP (*P* = 0.062) and decrease albumin levels (*P* = 0.055) by the end of CCl_4_ exposure (on day 28), but these differences were no longer evident by day 42 (*P* > 0.1). Total protein was also affected by CCl_4_ exposure, showing a slight decrease by day 42 (*P* = 0.055), while no difference was found on day 28. The rest of the parameters were not affected by the treatments applied.

**Table 3:** Effect of CCl_4_ and additive used on biochemical parameters^1^ at 28 and 42 d.

		AST (U/L)		ALT (U/L)		ALP (U/L)		GGT (U/L)		TP (g/dL)		Alb. (g/dL)	
		28 d	42 d	28 d	42 d	28 d	42 d	28 d	42 d	28 d	42 d	28 d	42 d
*Challenge*	*Additive*												
CCl_4_ 0 mL/kg BW	None	228	261	10^b^	77	17888	3816	18.3	22.6	2.39	3.0	0.50	1.0
	Silymarin	314	196	44^a^	66	13177	6269	20.5	18.9	2.30	2.9	0.79	0.8
	CA-PFA	230	123	35^a^	34	12649	5518	21.4	18.5	2.16	2.8	0.84	0.9
CCl_4_ 1 mL/kg BW	None	251	110	8^b^	41	13765	6456	18.9	18.9	2.25	2.6	0.65	1.0
	Silymarin	266	209	9^b^	48	25523	5105	19.3	21.0	2.28	2.8	0.55	0.8
	CA-PFA	253	232	9^b^	74	21950	5345	21.0	22.4	2.38	2.7	0.58	0.9
	*SEM (n = 8)*	*83*	*131*	*17*	*45*	*10535*	*3065*	*3.8*	*7.67*	*0.35*	*0.31*	*0.20*	*0.15*
*Main effects*													
CCl_4_	0 mL/kg BW	257	193	29^a^	59	14571	5201	20.0	20.0	2.28	2.9	0.71	0.9
	1 mL/kg BW	256	184	9^b^	54	20413	5635	19.7	20.7	2.30	2.7	0.59	0.9
	*SEM (n = 24)*	*84*	*137*	*19*	*46*	*10811*	*3051*	*3.82*	*7.52*	*0.35*	*0.30*	*0.23*	*0.16*
Additive	None	239	186	9^b^	59	15826	5136	18.6	20.7	2.32	2.8	0.58	1.0
	Silymarin	290	203	26^a^	57	19350	5687	19.9	19.9	2.29	2.8	0.67	0.8
	CA-PFA	241	177	22^ab^	54	17299	5431	21.2	20.4	2.27	2.7	0.71	0.9
	*SEM (n = 16)*	*82*	*138*	*21*	*46*	*11240*	*3084*	*3.70*	*7.60*	*0.35*	*0.32*	*0.23*	*0.14*
Effect (*P*-value)													
CCl_4_		0.974	0.798	<0.001	0.731	0.062	0.626	0.775	0.732	0.871	0.054	0.055	0.847
Additive		0.160	0.852	0.014	0.948	0.640	0.879	0.158	0.954	0.921	0.806	0.187	0.016
CCl_4_ x Additive		0.382	0.205	0.022	0.052	0.074	0.203	0.794	0.353	0.367	0.328	0.101	0.837

a–b Means in the same column with different superscripts are different (*P* < 0.05)

1 AST: Aspartate Amino Transferase; ALT: Alkaline Amino Transferase; GGT: Gamma Glutamyl Transferase; ALP: Alkaline Phosphatase; TP: Total Protein; Alb: Albumin

## DISCUSSION

Productivity in the poultry industry strongly relies on the return on feed investment and an adequate diet valuation, essential aspects for maximizing profitability and ensuring production sustainability. After the implementation of restrictions and bans on the use of antibiotic growth promoters in different countries (Rahman et al., 2022), adding PFA into the diet has become a frequently used strategy to boost production. Many commercially available products aiming to improve broiler productive performance have been developed based on documented benefits of different PFA. However, the doses used in most *in vivo* studies reported in the literature can be up to ten times greater than commercial suitable doses recommended in the poultry industry (Alagawany et al., 2022; Zaker-Esteghamati et al., 2020; Rahman et al., 2022; Surai and Surai, 2023; Sureshbabu et al., 2023).

The present work studied the influence of CA-PFA (inaplus3), a mixture of feed additives including milk thistle extract (silymarin), turmeric extract, and betaine hydrochloride. This product is recommended by the supplier to improve productive performance. Several *in vivo* studies that evaluate the efficacy of CA-PFA components individually show that, to detect a statistically significant improvement in productive performance, the required doses of each component are 200 mg/kg of feed or higher (Rajput et al., 2013; Sakomura et al., 2013; Armanini et al., 2021; Yang et al., 2022; Shanmugam et al., 2022; Surai and Surai, 2023; Sureshbabu et al., 2023; Zaki et al., 2023). The CA-PFA recommended commercial dose is 250 to 500 mg/kg of feed, which results in a smaller dose of its individual components than the reported efficacious dosages. In this work, we explored the CA-PFA effect on productive performance at the commercial recommended dose, under standard production conditions, and when the birds were exposed to hepatic damage.

The liver has a vital role in metabolism, and a suboptimal hepatic function impairs digestion and absorption of nutrients, causing a decrease in productivity. Hepatic damage is a prevalent issue in the poultry industry as a result of undesirable exposure of broilers to hazardous substances such as heavy metals, mycotoxins, and pesticides (Rozenboim et al., 2016; Haque et al., 2020; Guerrini and Tedesco, 2023). These conditions are challenging to recreate for *in vivo* trials; therefore, different strategies have been developed previously to induce and study liver damage. We selected low doses of CCl_4_ to chemically induce liver damage because this experimental model is a well-studied practical method that causes hepatic toxicity without atrophying metabolism (Rao et al., 1997; Weber et al., 2003; Sonkusale et al., 2011). After oral administration, CCl_4_ is concentrated in the liver and reaches a maximum level of about 1 mg/g of liver within 1 to 2 h of dosing (Reynolds, 1963). Histological evidence of tissue derangement occurs 5 to 6 h after administration, when necrosis begins (Lockard et al., 1983). Depending on the dose of CCl_4_, exposure time, presence of potentiating agents, or age of the affected organism, initial hepatocellular damage is followed by a period of repair that can lead to full recovery from liver damage (Rao et al., 1997; Weber et al., 2003; Sonkusale et al., 2011).

The detrimental influence of CCl_4_ in this experiment had a strong impact on productive performance and histopathological samples of the liver, mainly showing lipid accumulation, inflammation, and congestion of the liver, in accordance with previous reports (Sonkusale et al., 2011; Tsai et al., 2015; Baradaran et al., 2019). On the other hand, biochemical parameters did not show significant changes (*P* > 0.05), while other authors have reported an increase in serum enzyme activities (Wang et al., 2013; Joseph et al., 2014) and a decrease in protein and albumin levels (Sonkusale et al., 2011; Baradaran et al., 2019). However, in agreement with our results, some previous studies did not find significant differences in these biomarkers for hepatic damage (Tsai et al., 2015; Surai and Surai, 2023; Guerrini and Tedesco, 2023). In our case, the absence of significant differences could find an explanation in the previous work of Mehendale et al. (1991, 1996), who pointed out that CCl_4_ elicits 2 opposing responses, tissue repair and tissue damage, where the latter can overpower the former with bolus doses of more than 2 mL/kg BW in the rat (Rao et al., 1997). With low doses of CCl_4_, hepatocellular recovery will begin several hours after administration of the toxicant, approximately at the same time when centrilobular necrosis becomes evident (Mehendale, 1991; Calabrese and Mehendale, 1996). Also, Wang et al. (2013) studied the influence of different doses of CCl_4_ on liver function and detected a dose-dependent effect of CCl_4_ on ALT and AST levels, finding a significant increment in serum concentrations of these enzymes only in doses superior to 4 mL/kg.BW of a CCl_4_ peanut oil solution 1:1. The CCl_4_ dose applied in this study (1 mL/kg BW) impaired liver function, showing a detectable effect on performance parameters and liver histopathology. However, changes in biochemical parameters were not large enough to be significant under this experimental design.

Besides CCl_4_ exposure, we also examined the influence of CA-PFA and silymarin on productive performance and protective effects against liver damage. Silymarin has been extensively used to treat or prevent hepatic damage (Saeed et al., 2017; Baradaran et al., 2019; Armanini et al., 2021; Guerrini and Tedesco, 2023), and several studies have explored its benefits for human and veterinary use (Surai and Surai, 2023; Elnesr et al., 2023). However, specifically in the poultry industry, which demands practical and cost-effective solutions, the dose is a critical aspect to be analyzed when determining the efficacy of feed additives (Surai and Surai, 2023). Several authors have tested silymarin effects in ameliorating hepatic damage in broilers and obtained significant improvements in growth performance using doses around 500 mg/kg or higher in the feed (Khatoon et al., 2013; Jahanian et al., 2017; Aziz et al., 2019). At lower doses, silymarin has a positive influence on productive performance, but when compared with the additive-free diet, the differences are not always statistically significant (Schiavone et al., 2007; Blevins et al., 2010; Sakamoto et al., 2018; Lukanov et al., 2019; Armanini et al., 2021; Shanmugam et al., 2022; Surai and Surai, 2023). These results are consistent with the fact that silymarin is required in large doses to achieve therapeutic plasma levels because of its low bioavailability, poor solubility, rapid degradation by gastric fluids, and low absorption rates (Giacomelli et al., 2002; Dixit et al., 2007; Woo et al., 2007; Di Costanzo and Angelico, 2019).

The CA-PFA used in this study is a combination of turmeric extract, silymarin, betaine, among other bioactive compounds. In the present study, CA-PFA improved production performance parameters more efficiently than an equivalent dose of silymarin alone. During the experiment, no significant differences (*P* > 0.05) in FI were found in any treatments analyzed. Therefore, no differences in nutritional supply can be assumed, allowing us to correlate the effects detected in BWG and FCR mainly to the influence of the factors analyzed (exposure to CCl_4_ and additives used). Interestingly, the group treated with CA-PFA showed a steady tendency to increase BWG and reduce FCR, indicating that the weight increase is not the mere result of more vigorous birds consuming more feed but rather due to the reduction of the detrimental effect of the administered CCl_4_.

Neither silymarin nor CA-PFA showed the ability to significantly influence biochemical parameters that are indicators of liver damage, like ALT and AST, which have been previously recommended as biomarkers of hepatic damage in birds (Diaz et al., 1999; Ferrell, 2002). This could be a direct result of the low dose applied of the bioactive compounds in both treatments (Schiavone et al., 2007; Blevins et al., 2010; Sakamoto et al., 2018; Lukanov et al., 2019). Nevertheless, it is noteworthy that despite the low CA-PFA dose used, this product was able to mitigate CCl_4_ toxic effects and restore the growth performance of the birds. These results indicate that CA-PFA action is not due to the silymarin effect alone but rather to the complementary combination of all the ingredients in the formulation. Previous studies have obtained similar results when using formulated silymarin instead of standard silymarin products (Giacomelli et al., 2002; Passerini et al., 2012; Méndez-Sánchez et al., 2021).

Based on our results, the CA-PFA effect cannot be attributed only to the hepatoprotective properties of its ingredients because, qualitatively, CA-PFA improved liver health, but the differences found are not large enough to explain the improvement in productive performance. Additionally, CA-PFA improved performance for healthy birds, indicating that this product has more benefits than just improving liver health. It could be related to improved bioavailability of its components or nutrient digestibility, which could be explored in future studies. CA-PFA impact on productive performance could be the result of not just hepatoprotective activity but its combination with other benefits like anti-inflammatory, antimicrobial, antioxidant, and antistress properties that CA-PFA components present (Yarru et al., 2009; Akhavan-Salamat and Ghasemi, 2016; Nawab et al., 2020; Gana et al., 2023). CA-PFA may improve broilers’ overall health and well-being, translating into better productive performance.

## CONCLUSION

In summary, in the present study, CA-PFA improved the productive performance of birds with and without induced liver damage more efficiently than standard commercial silymarin. CA-PFA effect was particularly evident in BWG and FCR. CA-PFA did not significantly improve the hepatic parameters evaluated. Therefore, the hepatoprotective action of the active compounds is not the only factor at play when analyzing how CA-PFA improves productive performance parameters. Supplementing feed with CA-PFA could be an effective strategy to improve growth parameters in broilers.

## Appendix Supplementary materials

Image, application 1

## Footnotes

Supplementary material associated with this article can be found in the online version at doi:10.1016/j.psj.2024.103567.
