# Supplementary material for: Evaluation of commercial doses of a feed additive and silymarin on broiler performance with and without CCl4-induced liver damage
Source: Poult Sci. 2024 Feb 18;103(5):103567. doi: 10.1016/j.psj.2024.103567 (PMC10909905; doi:10.1016/j.psj.2024.103567)
Supplement: Supplementary file 1 [file mmc1.docx]

**SUPPORTING INFORMATION**

**Evaluation of commercial doses of a feed additive and silymarin on broiler performance with and without CCl4-induced liver damage**

Mabel Barreiro Carpio^[[1]](#footnote-1),†,1^, M. Alejandro Valdes-Pena^*,^^[[2]](#footnote-2)^, Daniel A. Molina^*^, Sandra E. J. Espinoza Cabello^*^, Carlos A. Sialer Guerrero^*^, Giovanna Cribillero^[[3]](#footnote-3),^^[[4]](#footnote-4)^, Katherine F. Vargas Coca^‡^ and Eliana Icochea^‡^

| **Table S1:** Growth performance before challenge | | | | |  |  |  |  |
| --- | --- | --- | --- | --- | --- | --- | --- | --- |
| **Additive** | **BWG (g)** | |  | **FI (g)** | |  | **FCR (g/g)** | |
|  | 7d | 14 d |  | 7d | 14 d |  | 7d | 14 d |
| None | 105 | 375 |  | 136 | 528 |  | 1.29 | 1.41 |
| Silymarin | 106 | 373 |  | 134 | 523 |  | 1.27 | 1.40 |
| CA-PFA | 104 | 377 |  | 134 | 525 |  | 1.29 | 1.39 |
| *SEM (n=16)* | *4.2* | *12.7* |  | *7.1* | *14.3* |  | *0.078* | *0.046* |
| ***P-Value*** | 0.39 | 0.65 |  | 0.69 | 0.68 |  | 0.49 | 0.62 |

| **Table S2:** Effect of challenge and hepatoprotective agent used on liver relative weight ^1^ | | | | | |
| --- | --- | --- | --- | --- | --- |
|  |  |  | **Liver RW (%)** | |  |
|  |  |  | 28d | 42d |  |
|  | ***CCl4 dose*** | ***Additive*** |  |  |  |
|  | 0 mL/kg BW | None | 2.40 | 2.25 |  |
|  |  | Silymarin | 2.34 | 2.18 |  |
|  |  | CA-PFA | 2.34 | 2.12 |  |
|  | 1 mL/kg BW | None | 2.72 | 2.49 |  |
|  |  | Silymarin | 2.78 | 2.48 |  |
|  |  | CA-PFA | 2.73 | 2.31 |  |
|  |  | *SEM (n=8)* | *0.208* | *0.237* |  |
| ***Main effects*** | |  |  |  |  |
|  | CCl4 | 0 mL/kg BW | 2.36 | 2.18 |  |
|  |  | 1 mL/kg BW | 2.74 | 2.43 |  |
|  |  | *SEM (n=24)* | *0.201* | *0.237* |  |
|  |  |  |  |  |  |
|  | Additive | None | 2.56 | 2.37 |  |
|  |  | Silymarin | 2.56 | 2.33 |  |
|  |  | CA-PFA | 2.53 | 2.22 |  |
|  |  | *SEM (n=16)* | *0.283* | *0.262* |  |
| ***Effect (P-Value)*** | |  |  |  |  |
|  | CCl4 |  | <0.001 | <0.001 |  |
|  | Additive |  | 0.922 | 0.185 |  |
|  | CCl4*Additive |  | 0.734 | 0.781 |  |
| ^1^ Liver RW: liver relative weight | | | | | |

1. R&D Department, Ilender Perú S.A., Lima, Peru [↑](#footnote-ref-1)
2. Current position: Department of Chemistry, NC State University, USA [↑](#footnote-ref-2)
3. School of Veterinary Medicine, Universidad Nacional Mayor de San Marcos, San Borja, Lima, Peru [↑](#footnote-ref-3)
4. Current position: Department of Poultry Science, Mississippi State University, USA

   ^1^Corresponding author: [mbarrei@ncsu.edu](mailto:mbarrei@ncsu.edu) [↑](#footnote-ref-4)
